# COVID-19 impact on mental health, healthcare access and social wellbeing – a black community needs assessment

**DOI:** 10.1186/s12939-022-01743-z

**Published:** 2022-09-22

**Authors:** Olihe Okoro, Elyse Carter Vosen, Kay Allen, Janet Kennedy, Renee Roberts, Taiwo Aremu

**Affiliations:** 1 University of Minnesota, College of Pharmacy, Duluth, MN USA; 2 Department of Global, Cultural, and Language Studies, The College of St. Scholastica, Duluth, MN USA; 3 Duluth, MN USA; 4 Healthy Alliances Matter for All LLC, Duluth, MN USA; 5 University of Minnesota, College of Pharmacy, Minneapolis, MN USA

**Keywords:** COVID-19, black/African American, Mental health, Healthcare access, Social well-being

## Abstract

**Background:**

The COVID-19 pandemic has had a disproportionate effect on the Black/African American population. In addition to the higher infection rates and the worse outcomes, there were other unintended consequences of the pandemic. The study objective was to determine the impact of COVID-19 on the Black/African American community.

**Methods:**

A needs assessment was conducted using a mixed-methods approach. To address this specific study objective, an item included in the survey questionnaire asked respondents (*n* = 183) about their greatest worry related to CODID-19*.* Interviews and focus group discussions were conducted to further explore individual and community perceptions.

**Results:**

The areas of greatest concern were Health (41.0%), Family (25.1%), Finances (8.2%), and Education (4.9%). The needs assessment revealed that the COVID-19 pandemic had a profound impact on the mental health and wellness, healthcare access and utilization, and social aspects of life the Black community. Emerging themes revealed that there was worsening mental health for many, limited healthcare access and under-utilization, and profound disruption of the social cohesive identity of the Black/African American community.

**Conclusion:**

Pre-existing structural inequities are implicated in the mental health impact, as well as the under-utilization of and limited access to healthcare services in the Black/African American population. The impact on social well-being emphasizes the important role of culture in the population health of communities of color, further supporting the need for culturally-responsive public health interventions when targeting these communities.

## Background

The Black/African-American population accounted for 12.4% of people living in the US in 2020 [1]. In Minnesota, the Black/ African-American population comprised 6.9% of those residing in the state at the time, making it the 2nd largest population by race [1]. The Black/African-American population in the United States has been disparately affected by the COVID-19 pandemic. There is evidence of racial disparities in COVID-19, with the Black/African American and Hispanic populations experiencing higher rates of SARS-CoV-2 infection and COVID-19–related mortality [2–5]. In the state of Minnesota, racial disparities in COVID-19 became more profound when age-adjusted excess mortality rates were taken into account [6]. Also apparent from the literature is the implication of social determinants in the COVID-19 racial disparities nationally and at the state level [5–7].

The intersection of the pandemic and social determinants precipitated other unintended consequences on communities of color, including challenges related to mental health, healthcare access and social wellbeing [8]. The pre-existing racial inequities in socioeconomic status and economic opportunity, along with other factors, not only increased risk of COVID-19 infection in communities of color, but were detrimental to other health outcomes [9]. Racial disparities in health care and mental health access adversely affecting the Black population was further exacerbated by the pandemic [10]. The fight against COVID-19 must therefore encompass means of addressing the indirect effects. Interventions can only be effective when they are informed by an understanding of these effects and the mechanisms by which they occur in the more vulnerable populations of color.

The study objective was to determine the impact of COVID-19 on the Black/African American community. Reported below are the effects related to mental health and wellness, healthcare access and social well-being.

## Methods

One of the aims of the study was to assess impact and identify priority areas of need related to the COVID-19 pandemic for the Black/AA community. Since COVID-19 was novel and the impact of great consequence, a needs assessment was proposed. This approach allows for identification of needs and action steps to address the needs identified.

### Data collection

A needs assessment was conducted using a mixed-methods approach. A web-based survey developed by the investigators was administered by disseminating the survey link through electronic and social media. The survey was active for a 3-month period (August 24 – November 28, 2020). Eligibility criteria for participation in the survey and interviews included being 18 years and older, and self-identifying as a person of African heritage (black/African-American) or bi−/multi-racial with black/African-American as one of their racial identities. The survey was built on the Qualtrics® platform to ensure compatibility with both computers and cell phones. A QR code was generated to facilitate non-contact dissemination and ease of access for safety per COVID-19. One of the items included in the survey asked respondents about their greatest worry related to CODID-19.

Thirty one-on-one interviews and eight focus group discussions (FGDs) were conducted with community members via Zoom. Interview respondents were different from FGD participants. Recruitment for interviews and FGDs was through community social networks and facilitated by community partners. Sampling was purposeful and ensured representation across categories of sociodemographic characteristics. Teenagers less than 18 years were included in two focus groups with consent from parent/guardian*.* A question on the semi-structured guide used to conduct the interviews and FGDs explored individual and community perceptions of the impact of COVID-19. Interviews lasted 19–48 minutes, while FGDs lasted between 53 and 135 minutes. All the interviews and focus groups were audio-recorded and transcribed. All participants gave written informed consent following a review of the study information at the point of recruitment prior to the interview or FGD, respectively. The study was approved by the University of Minnesota Institutional Review Board.

### Data analysis

Responses to the survey item related to the impact of COVID-19 were summarized using frequency counts and percentages of the response categories. A thematic analysis of the interview and focus group data was conducted with an initial coding of three transcripts completed by three independent investigators. Reconvening, these investigators reconciled differences in codes generated and came to consensus on codes, resulting in a coding sheet that served as reference for the coding of subsequent manuscripts. Additional codes were included when none of the prior codes captured new information. These codes were grouped into categories and corresponding excerpts pulled into respective excel spreadsheets per category. All coded data related to impact of COVID-19 was further analyzed and synthesized by two additional researchers. From this secondary analysis emerged themes that capture the perceptions of participants on the impact of COVID-19. In this report are themes specific to mental health and wellness; health services utilization and access; and impact on social aspects of life.

## Results

### Survey responses

There were 183 survey respondents, with ages ranging from 18 to 81 years. Approximately half of the respondents were female (49.7%) and 82.5% had some form of health insurance (see Table 1). The areas of greatest concern were Health (41.0%), Family (25.1%), Finances (8.2%), and Education (4.9%).

**Table 1:** Demographic characteristics of study participants

	Survey [*n* = 183	Interviews [*n* = 30]	Focus groups [*n* = 49]
	Frequency (percentage)		
**Race/Ethnicity**			
*African-American*	120 (65.6)	29 (96.7)	32 (65.3)
*African (+Jamaican)*	40 (22.9)	1 (3.3)	12 (24.5)
*Bi/Multi-racial*	23 (12.5)		4 (8.2)
**Sex**			
*Male*	90 (49.2)	14 (46.7)	23 (46.9)
*Female*	91 (49.7)	16 (53.3)	26 (53.1)
*No response*	2 (1.1)		
**Education**			
Middle/*High School*	64 (35.0)	13 (43.3)	14 (28.6)
*College (+ some credit)*	100 (54.6)	15 (50.0)	31 (63.2)
*Grad Studies*	17 (9.3)	2 (6.7)	3 (6.1)
*No response*	2 (1.1)		
**Employment**			
*Unemployed*	N/A	12 (40.0)	18 (36.7)
*Employed (PT)*		7 (23.3)	14 (28.6)
*Employed (FT)*		8 (26.7)	8 (16.3)
*Self-employed*		3 (10.0)	2 (4.1)
*Retired*			6 (12.2)
**Marital Status**			
*Single (Never Married)*	N/A	18 (60.0)	36 (73.5)
*Married*		4 (13.3)	9 (18.4)
*Divorced*		5 (16.7)	1 (2.0)
*Widowed*		3(10.0)	3 (6.1)
**Age**			
*Mean*	43.4	45.1	32.5
*Median*	42.0	44.0	21.0
*Range*	18–81	24–66	13–80

### Interviews and focus groups

The needs assessment revealed that the COVID-19 pandemic had a profound impact on the mental health and wellness, healthcare access and utilization, and social aspects of life of the Black community. Below are themes reflecting participants’ reports on these areas.

## Mental health and wellness

*1a. Worsening mental wellbeing:* The lockdown associated with COVID-19 necessitated that individuals stay indoors more often. Some of the participants linked this restriction to the experience of waning mental wellbeing, citing increase in stress, fatigue, anxiety, and depression among community members.

*They’re not happy. There’s a lotta depression. People stay in the house. Don’t wanna be bothered. Socially, everybody’s just withdrawn and afraid of each other. They don’t reach out for each other or talk to each other. It’s really depressing, I feel like. People’s spirits are dropping. They are mostly afraid of contacting the COVID by being around other people -----* [Interview Participant #14].

*I feel like COVID has affected people’s mental health a lot. I feel like a lot of people have a lot of anxiety and just, I feel like, depression too just because people have been forced to stay home, and so they’re used to not interacting with people when they have to quarantine and stuff*. [Participant, FGD #7].

Others acknowledged that the limited social life and isolation resulted in further deterioration of pre-existing mental health condition.

*I know that it’s affecting a lot of people, as well as myself, in the capacity of depression and stuff like that. Me, myself, having mental issues, I notice that I have to get to the gym room more because my anxiety and my depression is bad. [Participant #30].*

*I’m very isolated. If you wanna know the truth, I am getting depressed, but I know the symptoms of depression, so I’m staying aware like, “This is just my depression and anxiety that’s kicking in.* [Interview Participant #18].

*1b. Enhancement of mental wellbeing:* One participant reported that the lockdown did facilitate improvement of mental wellbeing particularly with regards to recovery from drug and alcohol abuse.

*For me personally, COVID has almost been a blessing. That’s very personal to me because of the timing of my recovery and trying to get clean from drugs and alcohol.* [Interview Participant #24].

Others saw it as an opportunity to spend more time with immediate family members and said it also provided more free time to engage in things outside work that they would not have otherwise. This included caring for the homeless, which contributed to improving the community health status.

*Busy. Just doing different little activities with the kids. Picking myself up and saying you know what? Let’s try to go outside today. Let’s do something today. Let’s play a board game today. Let me call my mom. Let me call a family member. Let’s talk about good memories, to put me in a good space. It has taken a toll some days*. [Interview Participant #16].

*To some extent, I see people of color that have more freedom because the virus has shut everything down. To some extent, if you’re in a situation where you can’t work because of the virus where—yeah, your employment—your hours have been cut, that gives people more free time. It gives them time to engage with other people in ways that they wouldn’t normally.* [Participant, FGD #4].

*1c. Associated stressors:* Participants commonly acknowledged that the pandemic increased stress levels both directly and indirectly. One major stressor was the fear of contracting the COVID-19 virus. Many participants reported incessantly worrying about high-risk family members, particularly, those in multi-generational homes with a vulnerable family member(s). Persons perceived to be at high risk included the elderly, those with underlying medical conditions and those engaged in jobs that brought them in constant contact with others.

*lotta my family has underlying health conditions. It was dangerous for me to be around them. It was a lot of isolation for myself, which wasn’t really the best for my mental health. Yeah, so I would say it was very, very, very detrimental, −--- for the Black community.* [Participant*,* FGD #1].

*I live with my grandma to help take care of her who she is 85, and so I’ve had to change a lot of my lifestyle as far as who I’m in contact with. I have to ask different people who I’m seeing who they’ve been in contact with, what they’ve been doing to keep themselves and others safe because obviously, she’s at a higher risk because of her age, and she also has some heart issues. Then because my mom works in the healthcare field, she also has to be more careful. My brother works at [organization] doing security. He has to be really careful.* [Interview Participant #12].

Many mentioned the enormous stress brought on by the loss of jobs, and consequently income, resulting in the inability to provide food, shelter, and other utilities.

*Yeah. I’ve seen my fair share of a lotta different things and ways. Some parents can’t afford the cable and the internet for their children anymore because they no longer working’, so they don’t have the extra income. Now, their children are outside, trying’ to find food.* [Interview Participant #16].

*Unemployment takes you through the ringer also. Every area of our low-income and marginalized people are impacted by this virus.* [Participant, FGD #4]

1. Healthcare Access & Utilization

*2a. Lack of insurance coverage pre-pandemic:* Participants emphasized the impact of lack of insurance coverage due to the prevalence of poverty in communities of color. They reported that with COVID-19, persons who did not have insurance prior did not necessarily assume that services would be free, especially as there were also reports of persons being billed for COVID-related services like testing in some facilities. This further discouraged people from seeking care as needed.

*“.*. *. one barrier—everything always goes back to financials, where people don’t have insurance to cover their expenses, so they have to pay with their own money and that seems very extreme. Bills are very expensive, especially hospital bills, so that discourages them from going there.”* [Participant, FGD #2].

*“There are some testing places that you have to pay for testing, or you need insurance and stuff like that. I feel like a lot of people can’t really afford getting tested, or they don’t have insurance. I feel like they need more options for getting free testing.”* [Participant, FGD#6].

*2b. Racism in healthcare:* Another limiting factor to utilizing healthcare services that came up was the experiences of racial discrimination, both historically and in contemporary settings/contexts. Some participants cited historical events that demonstrated long-standing systemic racism in healthcare while others referred to personal experiences with seeking medical care.

*“..*. *if you get into the medical care system here as a black person, you are vulnerable. I think people just wouldn’t—some people just wouldn’t seek care on that basis because of the history. We all know about how experimentation was done on us. ..* [Participant, FGD #5].

*“I think people underestimate the way you’re treated when you go into these health facilities. I always say racism happens every day. When you go anywhere, the way you’re treated can ruin your entire day. People forget that. Being at a cashier, or being at the place and they’re treating you like, yeah, like you’re less than a person, it would make me feel, damn, I don’t even wanna be here, let alone take this test with you, if you’re gonna treat me like that. I’ve actually been treated like that too. Not for COVID— People can make you feel like you’re not wanted.* [Interview Participant #15].

The anticipation of racial discrimination in the Healthcare system was a common theme in the discussions and was stressed as a deterrent to seeking medical care for COVID-19, which they believed contributed to the high morbidity and mortality rates in the Black population. One participant expressed a morbid fear of seeking medical care saying they would only do so under specific circumstances.

*Hospitals and doctors do not give the same type of care and treatment to black people that they do to other races just because they undermine the struggles that they’re going through and the pain, and they kinda brush it off until it’s too late. That’s the biggest problem I’ve seen with the healthcare, the health world, and then the black community. It’s like they don’t really care enough to help the black community*. [Participant, Interview #28].

*If somebody got the virus and they needed treatment and they went into the hospital and stuff, they’re not gonna get the same treatment that some other person might get treatment, if ya know what I mean. [Participant, Interview #22].*

*2c. Inequitable information access.* Some of the participants reported that the Healthcare system did not communicate effectively or in a responsive manner with the community regarding COVID-related services like testing. One of the primary reasons noted for underutilization of healthcare services in the Blacks/African American community was the inequitable access to information due to the digital divide consequent to socioeconomic circumstances. Black communities were thus generally less informed about healthcare services, compared to the majority white population, hence the under-utilization of COVID-19 related services.

*A lot of people don’t even know where to go or where to call. That’s just not something that’s information that’s just put out there all the time.----- That’s where the confusion lies, and whether or not they can get a free test if they don’t have insurance. That’s all connected to that. I don’t feel personally that the local hospitals have done enough to get out clear, concise information as to how people of low income or no health insurance can access the testing ------ The thing is that, you could have put it in the newspaper. The newspaper’s online now, and you have to subscribe. You know what I mean? That’s another problem* [Interview Participant #13].

*Lotta people don’t have internet service or either one of these iPhones and stuff to look up the resource of where these free places are to get help* [Participant, FGD #5].

One participant also alluded to disparities in the information flow to the white population versus the Black community, and literacy in healthcare service utilization.

*What I have found, living in this community, is white people’s information and other people’s information are so different. White people have so much more information than other people have. Unless you got a white friend, who shares that information with you, then you are in the dark. ---- white people would know immediately where to go, who to call, what to do, and I don’t know that the average person in our community would know the first steps. That’s a problem.* [Participant, FGD #3].

*2d. Transportation* ***:*** Participants reported that in the early months of the pandemic, access to sites for COVID-19 related services like testing was very challenging for members of the community who relied primarily on public transport.

*I think transportation is a big issue because people didn’t have money for like gas and things, and cars, like that, before. Just not having jobs, having limited resources and limited unemployment resources for that matter too, will probably limit people’s actual access to getting there. People would be like, yeah, I could probably take the test but how will I get there? I’d say transportation is probably something that was amplified due to COVID and due to people having to lose jobs*. [Interview Participant #15].

In the early months of the pandemic, COVID-19 testing by drive-through was also a deterrent for members of the community even when they had access to public transportation. One of the focus group participants narrated their own experience.

*Personally speaking, when I found out that my son had COVID, I had to tell my job. They gave me 24 hours to go get a COVID test. I don’t have a car. The bus that goes up to where they were doing the testing, the Mall, it’s right next to Walmart and stuff, that bus runs every two hours. I don’t even know. I had to call [name] to ask her to give me a ride there because by the time I got off of work at 5:00, the last bus had already left. That was a barrier for me. Then also, I don’t know if I actually made the bus if I would’ve been able to get tested because they came out to the car to do the testing.* [Participant, FGD #4].

There was acknowledgement that things did change over time to accommodate those without cars and sites opened up in spaces more accessible to the community.

2. Impact on Social Well-being

Impact on the social life of the community was a common experience at both the interpersonal and communal levels.

*3a. Disruption of existing relationships:* Participants repeatedly discussed the impacts of COVID-19 on their existing interpersonal relationships. Many reported intentional and purposeful isolation from family members for their own protection, especially those that were elderly or with underlying health conditions.

*Lotta my family has underlying health conditions. It was dangerous for me to be around them. It was a lot of isolation for myself, which wasn’t really the best for my mental health. Yeah, so I would say it affected our social life. We can’t really go out as much and see our family most of all. Especially if we have older family, we don’t want to let them be affected by coronavirus.”* [Participant, FGD#6].

Fear of interaction with family members or friends who were potentially asymptomatic carriers, reportedly led to distrust and isolation.

*It’s scary because when you think of COVID, you think of death or very ill and sick. It has hindered a lot of my relationships because you don’t know if you’re a carrier. Some people who have COVID, they are asymptomatic, and they don’t present with signs or symptoms. They can appear to be perfectly fine.”* [Interview Participant #16].

*3b. Limited opportunity for new relationships:* In addition to limiting existing relationships, opportunities for new relationships have also been hindered. Fear of exposure and mandated restrictions on gatherings limit the potential and desire to forge new relationships.

*“It’s changed my view on my social life. Now that I have to adapt to this new lifestyle, I’m less likely to go outside. I’m less likely to go hang out with different people, or meet different people, just because of the hesitation of not knowing if somebody might be exposed to it.”* [Participant, FGD#6].

*“Social life, people obviously can’t hang out with friends or get to know their peers or anybody else like we used to because of quarantine. Incoming freshmen won’t get the same—and won’t have that ability to meet new* people.” [Participant, FGD #2].

*3c. Dissociation from social networks:* Another source of reduced social connectivity was the dissociation from previously established social networks including those at church and the conventional in-person educational setting. The absence of peer connectivity and mentoring opportunities for both young adults and children in school was observed as a challenge.

*“... of the things that he had going for him was the fact that he was in school. He was with his peers. .. and that got taken from him. He’s suffered miserably behind it.”* [Participant, FGD#4].

*“it’s impacted our church in the sense we have not had—we’ve had virtual services since—I will say shortly after we started getting the—following the science, we stopped having church. It has affected us being able to o come around. It affects our social”* [Participant FGD #4].

*3d. Disruption of social life* **:** Events, parties, concerts, indoor dining, and family gatherings were canceled, or capacity restricted which drastically reduced the opportunities for social fulfillment. This disruption was often remedied by online video platforms, but several participants noted that this was not a sufficient alternative.

*“There’s some people, as I said, that won’t let you come over their house. You maybe can come over and see them from the car. There are no birthday parties. There’s no family reunions. There’s no gatherings. If there is, there’s so many individuals missing because as I said, there’s those that are okay with it and those that aren’t.”* [Interview Participant #25].

*“It stopped so many things for me, church, friends, the workout program I was going to, just the restaurants with—we always went out to fellowship after a lot of our meetings. We always go out to for food and coffee We don’t do that.”* [Interview Participant #19].

*3e. Decreased social support from established groups:* Decrease in social support was a common theme with many citing faith-based groups as their main source of community. The lack of social support in this context led to feelings of depression and disconnection from some participants.

*“Our church has been shut down for months. That brings a lot of depression. It really did.”* [Interview Participant #19].

*“The way we gather—we like to gather. Even church. Stuff like that. We weren’t able to communicate how we wanted to, be around each other how we wanted to, how we got to. I think that is part of our heritage”* [Interview Participant #20].

An additional lost social support group that several participants discussed was those linked to recovery from addiction to substance and alcohol use. They described this community as heavily reliant on each other for support in maintaining their recovery.

*“I still remained strong. I have weak, weak moments, and it’s been a lotta stress going through this, and I still maintain not only my recovery, but my sanity for the most part [laughter] those days, but it is so hard on some people. I did cry from time to time because I could not get to my people who would sustain some of what I’m doing in my life. We Zoom. We do Zoom, and we see each other.”* [Interview Participant #19].

*“I attend Narcotics Anonymous. I’m in recovery, and that’s really been the hardest, because us recovering addicts, we need each other and lean on each other*.” [Interview Participant #9].

*3 f. Increased community support:* While much of results thus far focused on decreased social support, both the interview and focus groups noted that they also saw an increase in community support. There were collaborations with non-profit organizations for food distributions, personal protective equipment, and other resources.

*“The community did come together. They were giving away a lot of free stuff, household cleaning stuff, laundry soap, soap to wash your body, food, tissue, diapers, and wipes for babies.”* [Interview Participant #2].

*“... the community and the church is trying to let people know that they can bring you food, or they could put a mask in the food box if you need mask.”* [Participant, FGD#5].

One participant felt a sense of unity within the black community during the pandemic. They explained that the black community watches out for each other and have mutual understanding that their community is at a higher risk for getting ill.

*I know that during this epidemic, us as a Black community came closer together and we stuck out our necks for each other, trying to make sure that we are all educated on the virus and making sure we’re all following the rules, trying to make sure nobody else tries to get us sick because even though—we are one of the most unprotected people in this country. It’s really important for all of us to watch out for each other and make sure we’re there for each other*. [Participant, FGD#2].

*3 g. Inaccessibility of social services:* Reduced accessibility of social services including mental health services, childcare, schooling, jobs, housing, and transportation were discussed extensively in both the interviews and focus groups. The restrictions placed by the government agencies that forced individuals to make appointments online further exacerbated the existing disparities for those who did not have access to internet or phones.

*“The other thing that I wanted to say is that government agencies also has put a lot of restriction. They’re lifting some of them now but—if you go down there, you don’t know how to make appointments. There’s no instructions and don’t even know if everybody have access to internet and even a cell phone. Communication period has been hampered by the restriction because of COVID-19, communication, in general, for a lot of people.”* [Participant, FGD#4].

Another participant discussed the compounding effect of these accessibility disparities by explaining how not having a car for transportation prevented them from receiving services from drive-through services, since many establishments closed their lobbies for safety considerations.

## Discussion

### Mental health and wellness

Prior to the COVID-19 pandemic, the Black/AA population had historically been disparately affected by mental health due to a variety of factors including socioeconomic factors and access to mental health services [10, 11]. Mental wellbeing has continued to deteriorate over the past few years among minority populations in the US, including Blacks/African Americans [12]. Our findings corroborate others that demonstrate that the COVID-19 pandemic worsened the pre-existing inequities in mental health [13]. Access to mental health services was further limited while pre-existing conditions were exacerbated by stressors related to health concerns and socioeconomic factors. The concerns expressed by participants related to risk of infection were not misplaced as populations of color continue to have higher prevalence of medical conditions that pose significant risk for higher COVID-related morbidity such as incidence and hospitalizations [14, 15].

An unanticipated finding was the acknowledgment of some of the gains of the COVID-19 pandemic. These included the ability to be cut off from public engagement, freeing up time for recreational activities and more connection with family members, which contributed to health and wellbeing. Improvements in mental health and continued sobriety were also attributed to poor access to routinely abused drugs and alcohol, and the opportunities for inward reflection during the lockdown. These positive outcomes related to substance use and alcohol are likely outliers as there is overwhelming evidence in the literature demonstrating that the pandemic worsened addictions and substance-use disorders especially for the Black/AA population [16, 17]. Findings from the current study support the data demonstrating higher rates of depression and other mental health conditions triggered by socioeconomic factors in populations with low income, low assets, and loss of jobs, which are historically communities of color [18].

### Healthcare access and utilization

Health insurance is a major facilitator of healthcare access. Disparities by race/ethnicity continue to exist in insurance coverage. The decrease in this gap following the implementation of the Affordable Care Act (ACA) has since stalled, with Blacks and other populations of color remaining more likely to lack health insurance compared to the white population [19–21]. Analysis of the healthcare expenditure in the US pre-pandemic, showed lower utilization of outpatient services and higher utilization of emergency room (ER) services among Blacks compared to the national average [22]. In the wake of the COVID pandemic, the loss of jobs and decrease in employment opportunities that disparately affected communities of color further disrupted health insurance coverage [19]. Our findings highlight how prior lack of insurance influenced health-seeking behaviors related to COVID-19.

Blacks/AAs continue to experience racial discrimination and the impact of structural racism in the healthcare system [23–26]. Racism has been shown to influence healthcare utilization among Blacks/AAs even when healthcare access is not a barrier [27–29]. Findings from the current study give credence to the fact that insurance coverage, while key to healthcare access, does not necessarily assure healthcare utilization in this population. In addition, the inequitable access to health-related information that emerged as a key theme points to structural inequities that continue to affect communities of color. Information sharing through avenues and media that low-income populations may not have access to or efficacy to navigate, clearly disadvantages these communities. Furthermore approaches that are conventional, but not culturally-responsive are often not effective in health education and communicating information to communities of color [30, 31]. Poor communication to Black/AA populations has perpetuated the historical distrust of the healthcare system and medical research community [32–34].

These barriers associated with healthcare access fostered hesitancy in utilizing healthcare resources related to COVID-19, which were readily available and at no cost. This pattern of health-seeking behavior likely added to the burden of COVID morbidity and mortality in this population. Higher mortality in the Black/AA population for several medical conditions have been attributed to late stage presentation due to less than optimal utilization of preventive and regular care [35–37]. The historical trend appears to persist with COVID-19.

### Social well-being

As anticipated due to COVID-19 restrictions, decreased social connection was a common sentiment. Even so, this area is uniquely multifaceted and nuanced as not only were existing interpersonal relationships hindered, but also opportunities to make new connections. Prior successfully established support systems were also susceptible to experiencing disruption. Family and Church are significant sources of support for the Black/AA community [38, 39]. During the pandemic, churches and other religious gathering sites were closed with some not able to have services online. This led to a significant loss and social support, community engagement, including childcare, and resource/health information allocation. The significant changes in support affected mental health and wellbeing.

While some felt that they lacked sufficient support, others observed that the community was able to rally together to look out for and support each other. The importance of the Black community working together to distribute resources and health information is highlighted. Many cited the community and Church as providing resources and COVID-related information. The communal identity and trust are important and must be considered when providing support or collaborating with these communities.

## Recommendations

Recommendations from participants are summarized in Table 2. These are separated into the three broad themes of healthcare access, mental health and social impacts as previously categorized. The recommendations mostly address structural inequities that existed prior to the COVID-19 pandemic.

**Table 2:** Recommendations to address health and social impact

Mental Health and Wellbeing	Healthcare Access and Utilization	Social Well-being
• Community mental health clinics ○ Case managers as an option ➔individualistic approach to treatment ○ A buddy program. • Address socioeconomic challenges that exacerbate stress – employment, income, housing, etc.	• Establishment of a community-based health education program • Credible messengers who the community members trust and can easily access for information about their health and wellbeing • Provision of alternative options and support for individuals who are unable to utilize drive-through services or access online appointment scheduling.	• Increased emphasis on one-on-one instruction for students who are in online learning environments. • Expansion of opportunities for creating new support circles especially in school age children, people in recovery, and others who may need additional supports. • Reinforcement and maintenance of existing support circles. • Improvement or addition of socioemotional support and mental health education in schools.

## Conclusions

This study uncovers unexpected positive gains of the COVID-19 pandemic. These included the ability to cut off from public engagement, freeing up time for recreational activities and more connection with family members, which contributed to health and wellbeing. Another interesting find was improvement in mental health and continued sobriety for some community members, which was attributed to limited access to routinely abused drugs and alcohol, in addition to the opportunity the pandemic presented for inward reflection.

This study highlights the communal cultural identity of the Black/African American population whereby community health is often prioritized over individual health, hence the emphasis on the protection of the more vulnerable members (e.g. the elderly). The collective identity is also demonstrated in the uniting of community to meet the needs of members.

Pre-existing inequities are implicated in the mental health impact, as well as the under-utilization of and limited access to healthcare services. Worthy of note is how conventional communication channels used in dissemination of health-related information may further fosters disparities in healthcare access. The impact on social well-being emphasizes the important role of communal identity in the population health of communities of color, further supporting the need for culturally-responsive, public health interventions when targeting these communities.

## Data Availability

The survey data collected and analyzed during the current study are available from the corresponding author on reasonable request. The interview and focus group data (transcripts) generated from and analyzed during the current study are not publicly available. This is to protect community members’ privacy, as also stipulated in our study protocol.
